# Exploring Dietary Interventions in Autism Spectrum Disorder

**DOI:** 10.3390/foods13183010

**Published:** 2024-09-23

**Authors:** Ingrid Daniela Pérez-Cabral, Ariadna Thalía Bernal-Mercado, Alma Rosa Islas-Rubio, Guadalupe Miroslava Suárez-Jiménez, Miguel Ángel Robles-García, Andrés Leobardo Puebla-Duarte, Carmen Lizette Del-Toro-Sánchez

**Affiliations:** 1 Departamento de Investigación y Posgrado en Alimentos, Universidad de Sonora, Hermosillo 83000, SO, Mexico; a222230199@unison.mx (I.D.P.-C.); thalia.bernal@unison.mx (A.T.B.-M.); miroslava.suarez@unison.mx (G.M.S.-J.); a215201577@unison.mx (A.L.P.-D.); 2 Coordinación de Tecnología de Alimentos de Origen Vegetal, Centro de Investigación en Alimentación y Desarrollo A.C. (CIAD, A.C.), Hermosillo 83304, SO, Mexico; aislas@ciad.mx; 3 Department of Medical and Life Sciences, Cienega University Center (CUCIÉNEGA), University of Guadalajara, Av. Universidad 1115, Lindavista, Ocotlán 47820, JA, Mexico; miguel.robles@academicos.udg.mx

**Keywords:** autism spectrum disorder, nutrition, vitamins, oxidative stress, diets, food selectivity, gastrointestinal disorders

## Abstract

Autism spectrum disorder (ASD) involves social communication difficulties and repetitive behaviors, and it has a growing prevalence worldwide. Symptoms include cognitive impairments, gastrointestinal (GI) issues, feeding difficulties, and psychological problems. A significant concern in ASD is food selectivity, leading to nutrient deficiencies. Common GI issues in ASD, such as constipation and irritable bowel syndrome, stem from abnormal gut flora and immune system dysregulation. Sensory sensitivities and behavioral challenges exacerbate these problems, correlating with neurological symptom severity. Children with ASD also exhibit higher oxidative stress due to low antioxidant levels like glutathione. Therapeutic diets, including ketogenic, high-antioxidant, gluten-free and casein-free, and probiotic-rich diets, show potential in managing ASD symptoms like behavior, communication, GI issues, and oxidative stress, though the evidence is limited. Various studies have focused on different populations, but there is increasing concern about the impact among children. This review aims to highlight the food preferences of the ASD population, analyze the effect of the physicochemical and nutritional properties of foods on the selectivity in its consumption, GI problems, and antioxidant deficiencies in individuals with ASD, and evaluate the effectiveness of therapeutic diets, including diets rich in antioxidants, gluten-free and casein-free, ketogenic and essential fatty acids, and probiotic-rich diets in managing these challenges.

## 1. Introduction

Autism spectrum disorder (ASD), previously known as autism, is a complex developmental disability characterized by social communication difficulties and restrictive repetitive behaviors [1]. It is called a “spectrum” because it includes a variety of manifestations and degrees of severity. ASD symptoms typically appear in the first years of life and persist into adulthood, including cognitive impairment, seizures, sleep disturbances, gastrointestinal (GI) issues, feeding difficulties, and mood disorders [2]. Additionally, psychological problems like anxiety, depression, and behavioral challenges such as difficulties in communication, social interaction, resistance to change, attention and focus issues, and sensory sensitivities are also common [3].

In addition to autism, *The Diagnostic and Statistical Manual of Mental Disorders* (DSM-V) considers Asperger syndrome as a part of the spectrum of ASD. Asperger syndrome is a condition where intellectual levels are average or above average in terms of communication skills but with difficulties understanding social situations [4]. The DSM also considers as part of ASD a condition named childhood disintegrative disorder, which is a drastic loss of behavioral and developmental functioning after at least two years of normal development [5]. However, there are several classifications and levels of ASD severity.

The prevalence of ASD has increased significantly worldwide, with the World Health Organization estimating that approximately 1 in 100 children globally have autism [6]. In the United States, the Centers for Disease Control and Prevention (CDC) report that about 1 in 36 children are diagnosed with ASD [7], and in Mexico, the prevalence is approximately 1 in every 115 children [8]. This increase can be attributed to greater awareness, improved diagnostic methods, and changes in diagnostic criteria. However, prevalence varies considerably between regions and countries. Despite these variations, the global rise highlights the urgent need for resources and public health policies to support individuals with ASD and their families.

One of the main characteristics of ASD is an eating disorder frequently marked by strong food selectivity and pronounced dietary preferences [9]. This pattern is often linked to sensory sensitivities, which can be heightened (hypersensitivity) or reduced (hyposensitivity), affecting senses like touch, sound, light, and smell, depending on the individual [10]. These sensitivities frequently result in food neophobia (reluctance to try new foods) and dietary restrictions [11]. Neurodivergent individuals, including those with ASD, exhibit higher food selectivity, sensitivity to textures, brand-specific preferences, and feeding difficulties than their neurotypical counterparts [12,13]. This selectivity may lead to nutrient deficiencies and an imbalanced body composition, with tendencies toward underweight and obesity [10]. Individuals with ASD are considered neurodivergent, as this term refers to people whose neurological development differs from what is considered neurotypical (a person who does not have a neurological disorder) [14].

ASD is usually accompanied by GI issues that range from mild to severe symptoms, such as constipation, abdominal pain, diarrhea, and irritable bowel syndrome. The exact etiology of these issues is unknown; however, it is thought to be a mix of variables such as aberrant gut flora, immune system dysregulation, and increased intestinal permeability [15]. Furthermore, the sensory sensitivities and behavioral challenges associated with ASD can increase feeding difficulties, worsening GI symptoms. GI problems correlate favorably with the severity of neurological symptoms in persons with ASD, highlighting the importance of focusing therapies on these patients’ GI health [16]. In addition, children with ASD often exhibit higher oxidative stress caused by lower levels of antioxidants, such as glutathione (GSH), the main intracellular antioxidant crucial for combating oxidative stress and maintaining cellular health, as well as by mitochondrial dysfunction, increased lipide peroxidation, and other markers [17]. Oxidative stress has been linked to some neurological disorders, including ASD [18]. Antioxidant deficiencies in people with ASD may exacerbate symptoms such as behavioral disorders, cognitive impairments, and GI problems.

Treating these deficits with dietary modifications, supplements, and general nutritional support is becoming a more significant component of ASD management. Among the array of treatment options available, therapeutic diets have been somewhat under-researched despite their widespread adoption by families dealing with autism [19]. Several survey studies have highlighted the positive outcomes associated with therapeutic diets, showing improvements in specific ASD symptoms such as behavior, communication, and overall health. Additionally, benefits have been reported in managing GI problems, attention issues, communication skills, and social interaction [20,21]. Despite widespread interest in dietary interventions, there is no agreement on an effective nutritional therapy.

A primary motivation for implementing therapeutic diets is the observation that children with ASD frequently have restricted diets, often consuming only a limited variety of foods [22]. This tendency raises concerns about potential deficiencies in both macronutrients and micronutrients, underscoring dietary interventions’ importance [10]. Therefore, this review aims to highlight the food preferences of the ASD population, analyze the impact of food selectivity, GI problems, and antioxidant deficiencies in individuals with ASD, and evaluate the effectiveness of therapeutic diets, including diets rich in antioxidants, gluten-free and casein-free, ketogenic and essential fatty acids, and probiotic-rich diets in managing these challenges.

## 2. Methods

This narrative review evaluated the impact of food selectivity, GI issues, antioxidant deficiencies, and therapeutic diets on individuals with ASD. A comprehensive scientific literature search was conducted using the Scopus, PubMed, Web of Science, and Google Scholar databases. The search included articles and review articles written in English and published between 2000 and 2024 to ensure the inclusion of relevant and up-to-date research. The search terms used included “autism spectrum disorder”, “food selectivity in autism”, “gastrointestinal issues in autism”, “antioxidant deficiency in autism”, “therapeutic diets in autism”, “ketogenic diet in ASD”, “gluten-free diet in ASD”, “casein-free diet in ASD”, “antioxidant supplementation in autism”, “polyphenols and ASD”, “oxidative stress in ASD”, and “peptides in ASD”. Articles were selected based on their relevance to ASD, focusing on peer-reviewed studies, clinical trials, meta-analyses, and systematic reviews. The inclusion criteria comprised studies examining food selectivity in individuals with ASD, the link between GI issues and neurological symptoms, the role of oxidative stress in ASD, and the effectiveness of various dietary interventions. Articles unrelated to the key topics or lacking empirical data were excluded. The findings were synthesized to provide a comprehensive overview of the relationship between ASD symptoms and dietary interventions, highlighting current knowledge gaps and areas for further research. The review included literature with significant findings.

## 3. Nutritional Challenges in Autism Spectrum Disorder

Individuals with ASD encounter significant challenges in their nutrition, primarily stemming from their strict food preferences and difficulties in digesting and absorbing food [23]. Hence, several factors need to be taken into account before implementing any dietary intervention for managing ASD in individuals, including food selectivity, GI issues, and oxidative stress.

### 3.1. Eating Difficulties

Food selectivity is a developing health problem for children with ASD. Food selectivity can be defined as food rejection, food restriction, or the intake of a particular food [24]. Food selectivity, considered an eating disorder affecting 70–80% of children with ASD for over two years, negatively impacts health, nutrient intake, and family relationships, especially during meals [25]. To a certain degree, food selectivity and feeding disorders are observed in neurotypical children, but they tend to be more severe in individuals with ASD [26,27].

Sensory, behavioral, and biological factors influence food selectivity in individuals with ASD. Sensory sensitivities play a crucial role, as many children with ASD are hypersensitive or hyposensitive to specific characteristics of foods, such as the textures, tastes, colors, smells, appearance, and temperature of foods [28,29]. Sensory sensitivities in individuals with ASD vary widely, though specific patterns are observed. Commonly, textures like soft or mushy foods, strong tastes such as bitter or spicy, and strong smells are often rejected. In terms of colors and appearances, individuals may avoid foods or objects that are visually irregular or unfamiliar. Temperature preferences also play a role, with some rejecting foods that are too hot or cold. However, these preferences are highly individualized, meaning that sensory responses differ greatly from person to person, making it crucial to consider personal variability when addressing sensory challenges in ASD [30]. These sensitivities can make certain foods intolerable, severely limiting the variety of accepted foods. Furthermore, behavioral factors, such as restrictive and repetitive interests and actions, contribute to dietary selectivity [26].

Another feeding difficulty (not disorder) common in ASD individuals is food neophobia, manifested as a consistent refusal of foods with particular characteristics or a reluctance to try new foods, which may occur with varying frequency across individuals [31]. Refusing solid foods is common among individuals with ASD, and introducing foods with new textures, consistencies, and flavors often proves challenging [32]. As a result, they tend to consume the same foods repetitively. Children with ASD typically choose processed, sugary, and high-fat diets and reject fruits and vegetables [33,34]. Preferences for unhealthy foods can increase the risk of obesity and deficiencies in vitamins and minerals. 

Sensory sensitivity, particularly oral sensitivity, can partially explain food selectivity and food neophobia in ASD [31,35]. Studies have shown that children with ASD often exhibit atypical sensory processing, including a heightened sensitivity to taste, touch, and other sensory inputs [36]. This can lead to food rejection based on their sensory characteristics and contribute to persistent food refusal behaviors. Neurological factors in ASD may reinforce these behaviors, making them difficult to change. Early evaluation and intervention for these sensory-related eating issues are crucial to prevent malnutrition and other health problems related to inadequate nutrient intake [25,37,38].

In the case of ASD, these behaviors can significantly disrupt the child’s daily functioning and hinder their integration within peer groups [39]. Moreover, mealtimes can be especially challenging for people with autism and their families. Each meal can become a source of stress and frustration for the parents as they try to balance the nutritional needs of the individual with autism with their sensory and behavioral limitations [40]. This stress can affect family dynamics and make meals a tense and emotionally charged time. In addition to the understanding that introducing new foods may exacerbate food selectivity in individuals with ASD, it is recognized that inappropriate mealtime behaviors can contribute to restrictive eating habits and poor nutrition [41,42].

Food selectivity causes children with ASD often to have diets deficient in essential macronutrients and micronutrients, such as protein, vitamins, and minerals. This lack of nutrients can affect physical growth and development, leading to problems such as low weight, obesity, and deficiencies in the immune system. In the long term, these deficiencies can have serious health consequences, including the risk of chronic diseases and impaired cognitive and emotional development. For example, Molina-López et al. [10] conducted a cross-sectional case–control study in 144 children (*n* = 55 with ASD; *n* = 91 with neurotypical children) between 6 and 18 years of age to examine body composition, nutritional intake, food consumption frequency, and mealtime behavior. This study revealed that children with ASD showed a lower weight (18.4% ASD vs. 3.20% comparison group) and higher obesity (16.3% ASD vs. 8.6% comparison group) than neurotypicals. In addition, ASD children had a greater intake inadequacy (50% ASD vs. 22% comparison group), high food selectivity (60.6% ASD vs. 37.9% comparison group), and more eating problems (food rejection, limited variety, disruptive behavior) compared to neurotypical children. Children with ASD exhibit more altered mealtime behaviors, leading to higher food selectivity and nutritional deficiencies (moderate effect). Children with ASD reported a greater risk of nutritional insufficiency when exhibiting food selectivity [31]. According to Sharp et al. [43], 78.5% of children with ASD had an inadequate diet in terms of five or more nutrients. Early diagnosis enables parents and caregivers to conduct nutritional interventions on time, with the assistance of specialists. Some nutritional inadequacies have been related to the disorder’s pathophysiology; thus, addressing these concerns as soon as possible is critical to improving the health and well-being of those affected.

Table 1 shows different studies where various foods were evaluated by frequency of consumption by children with ASD. This information reveals that food acceptance levels for children with ASD varied significantly due to attributes such as texture, temperature, color, etc., and they predominantly accepted smooth and easy-to-chew foods [9,44,45]. Additionally, these children prefer foods with high sugar and avoid vegetables [46]. It also indicated that neurodivergent children, particularly toddlers with ASD, consume more snacks than neurotypical children, who tend to have a more diverse diet [47]. A survey showed that children with ASD who were picky about foods had more GI disorders than children without food selectivity [48]. Food selectivity may contribute to GI problems in individuals with ASD, as a limited diet can disrupt the balance of gut microbiota, increasing the risk of issues like constipation or irritable bowel syndrome [49]. Additionally, many individuals with ASD prefer processed foods, which can impair gut motility. Sensitivities or intolerances to certain foods may further trigger inflammation, leading to symptoms such as abdominal pain or diarrhea [50]. It highlights the importance and urgency of parents finding new snack options that are sensorily appealing and offer an adequate nutritional profile [51]. 

All these studies should be considered cautiously, as they present some limitations. For instance, many of them do not correlate the severity of ASD with the degree of food selectivity. Additionally, numerous studies compare children with ASD to independent neurotypical children, which means that the factors influencing differences in food selection could also be due to economic, familial, social, and other factors [52]. Therefore, studies involving neurotypical siblings could be particularly insightful, as the results of a few previous studies have shown [53,54,55].

### 3.2. Gastrointestinal Disorders

Apart from the range of symptoms observed in the primary diagnostic criteria, individuals with ASD may exhibit non-neurological comorbidities, such as problems related to the GI tract [50,56]. Children with autism have a high incidence of experiencing general symptoms related to their GI disorders, and, consequently, this might potentially influence food choices and contribute to instances of food refusal [57]. Esposito et al. [58] performed a questionnaire interview in a case–control study and found that GI symptoms, parenting style, and sensory abnormalities were linked to children’s food avoidance. In children with ASD, GI symptoms were related to a heightened sensitivity to moving visual stimuli and smell. In another study, ASD participants with feeding problems had a higher rate of GI symptoms and challenging behavior and sensory issues [59].

The prevalence of GI symptoms in the ASD population can range from 20 to 80% [60]. The most common GI problems in individuals with autism are chronic constipation, diarrhea, and abdominal pain [61], with elevated occurrences of gastroesophageal reflux, bloody stools, vomiting, gaseousness, and signs of GI inflammation; increased intestinal permeability, food allergies, altered dietary nutrient intake, and metabolic disruptions have also been linked to ASD [50,56]. In the systematic review carried out by Herrera-Mejía et al. [16], they found that the most prevalent GI symptoms were constipation (50–88.5%), flatulence (51–87.5%), abdominal distension (33–87.5%) and diarrhea (12.5–72.8%). In some studies, GI disorders were more prevalent and severe in ASD patients than in neurotypical volunteers.

**Figure 1:**
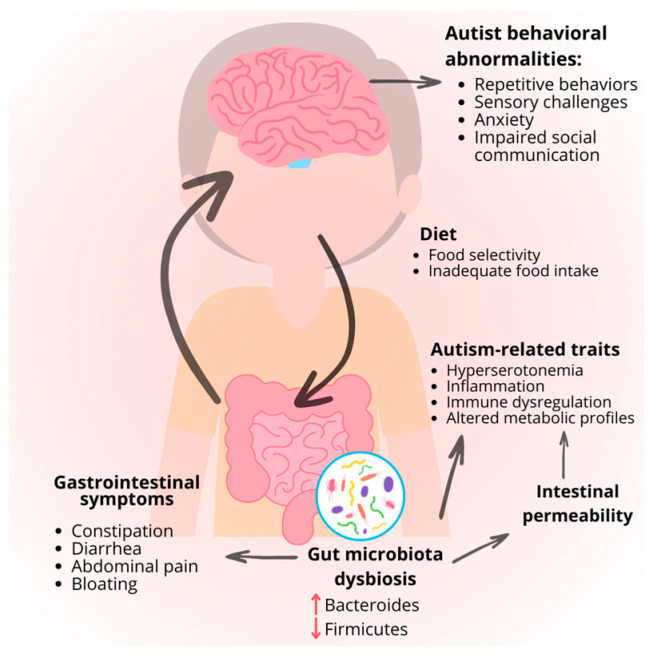
Impact of gastrointestinal symptoms on autism spectrum disorder symptoms. Figure created by the authors. ↑ means increase; ↓ means decrease.

The mechanisms of such disorders are not fully understood and appear to have a multifactorial basis, meaning they can arise from a combination of different factors [62]. In children and adolescents with ASD, GI problems are positively correlated with the severity of neurological manifestations (Figure 1) [55]. For example, compared to children with less severe ASD, those with more severe ASD experienced diarrhea 10% more frequently. Children with ASD are more likely than neurotypicals to have GI symptoms at least occasionally [63]. Despite some discrepancies, most studies confirm a significant correlation between GI problems and ASD compared to healthy, age-matched controls [62].

The pathophysiology of ASD may be influenced by the gut microbiota and its metabolites. Studies have demonstrated that, compared to neurotypical children, individuals with ASD exhibit a less diverse gut microbiota, a scarcity of beneficial bacteria, and a higher density of harmful bacteria [64]. Dysbiosis is widespread in ASD individuals, and particular abnormalities in the gut microbiota have been identified in patients with GI disorders [60]. The normal intestinal microbiota comprises beneficial bacteria such as *Bifidobacterium*, *Lactobacillus*, Bacteroidetes, and Firmicutes, which play crucial roles in digestion, producing short-chain fatty acids and immune system modulation. Maintaining a proper balance between these bacteria, particularly the Bacteroidetes/Firmicutes ratio and a high microbial diversity, is essential for intestinal health. An imbalance, such as an increase in Firmicutes and a decrease in Bacteroidetes, has been associated with metabolic disorders and inflammatory bowel diseases, among other conditions. 

The study by Shaaban et al. [65] found that the *Bifidobacterium* concentrations in children with ASD were lower than those in children without ASD. Similarly, Tomova et al. [55] reported that patients with ASD had a lower Bacteroidetes/Firmicutes ratio and higher levels of lactobacilli species compared to neurotypical volunteers. In the same study, patients with more severe GI symptoms had lower Bacteroidetes/Firmicutes ratios and a greater abundance of the bacteria *Clostridium* and *Desulfovibrio* than those with mild or moderate symptoms. The severity of the symptoms is correlated with the degree of GI microbial dysbiosis [61]. The systematic review by Nogay and Nahikian-Nelms [66] found that, among the analyzed studies, high growth rates of *Clostridium histolyticum*, *C. perfringens*, and *Sutterella*, a high ratio of *Escherichia*/*Shigella*, and a low ratio of Bacteroidetes/Firmicutes were generally related to GI problems. However, they concluded that the published studies on the relationship between GI and behavioral issues and the gut microbiota in autism are minimal and contradictory.

Changes in the microbiota accentuate neurological and digestive problems by stimulating responses to localized inflammatory processes that increase intestinal permeability [16]. There is increasing interest in the morphology of the GI structure in children with ASD, which can present a high intestinal permeability (nearly 40% in comparison to the neurotypical public, presenting only <5%), high levels of pro-inflammatory lymphocytes, or even colonic lesions due to epithelial damage [67,68]. GI problems in ASD patients, including increased intestinal permeability, altered gut microbiota, and dysregulated GI motility and secretion, can influence the development of other autism-related traits. These disruptions can affect serotonin production, leading to the association of hyperserotonemia with ASD. The increased intestinal permeability leads to the entry of bacterial metabolites or partially digested nutrients into the bloodstream, accentuating digestive, metabolome, and neurological disorders. These GI issues can also cause immune dysregulation, affecting behavior and brain function via the gut–brain axis through direct and indirect pathways involving the vagus nerve and immune system changes [56]. This can result in various effects, such as hyperserotonemia, where increased serotonin production in the gut raises blood serotonin levels, which is linked to repetitive behaviors in ASD. Additionally, altered gut microbiota, particularly an increase in *Clostridium* species, can influence anxiety, hyperactivity, and other behaviors through the gut–brain connection [56].

In a 2010 consensus report by Buie et al. [69], 23 statements were provided regarding children with ASD and various aspects of diagnosing and treating GI disorders in this population. Among these statements, it was suggested that a combination of behavioral treatment (such as identifying and communicating pain and teaching coping strategies for discomfort) and dietary manipulation (such as gluten-free and casein-free diets) could help reduce mealtime discomfort in some individuals. The report also emphasized the need for further research on nutritional interventions, with guidance from qualified nutritionists and pediatricians, while noting that dietary manipulation should not be used as the primary treatment for ASD.

### 3.3. Allergies

For food allergies, it has been reported it is 1.8 times more likely for ASD individuals to have some degree of asthma or food allergy. When put into comparison to the general pediatric population, around 5% of neurotypical children have food allergies, whereas in autistic children, this percentage is up to 20 to 25%. The systematic review and meta-analysis of Wang et al. [70] found that the prevalence of food allergy was higher in participants with ASD than in controls, and participants with ASD were at risk of developing food allergy. Food refusal could often indicate selectiveness over food and reflect a food allergy that has not been well explored or diagnosed [71].

The study of Alhuzimi and Alharbi [72] indicates that autistic children are allergic to proteins mainly. Indeed, serum levels of immunoglobulins IgA, IgG, and IgM specific for cow’s milk-derived allergens and total IgE were increased in children with ASD compared with healthy controls [73]. Allergies can also occur due to other foods such as peanuts, nuts, shellfish, meats, fruits, vegetables, grains, and seeds. Serious neurological abnormalities and other problems have been detected because when children with ASD have contact with the food allergen, cytokine signals increase, derived from the degranulation of mast cells and basophils induced by inflammation. Genetic and environmental factors in the prenatal and postnatal phases play a role in the development of allergic immune reactions in ASD individuals [74]. This still needs more research since many of the effects could disappear over time, mainly in adolescence. On the other hand, as many symptoms and illnesses can be confused with food allergies, it is important for parents to know the differences.

### 3.4. Oxidative Stress

Oxidative stress is a condition characterized by an imbalance between the production of reactive oxygen/nitrogen species (ROS/RNS) and the antioxidant defenses naturally found in organisms [75]. This is caused by excessive ROS/RNS or decreased synthesis of functional antioxidant protection capacity [76]. Fundamentally, ROS and RNS include superoxide (O_2_^−^), hydroxyl, peroxyl, alkoxy, hydrogen peroxide, and peroxynitrite free radicals. These molecules in high amounts can cause significant cell membrane damage, alterations in membrane fluidity and permeability, and induce oxidative changes in proteins, lipids, and DNA [77]. It has been reported that oxidative damage caused by free radicals plays a substantial role in the development, occurrence, and severity of many pathogenic diseases, including autism, Alzheimer’s, Parkinson’s, amyotrophic lateral sclerosis, diabetes mellitus, cardiovascular and inflammatory diseases, and cancer, among others [17]. Several factors contributing to the increase in oxidative stress are associated with contemporary lifestyle habits, such as an unhealthy diet, physical inactivity, smoking, and exposure to alcohol, pesticides, and food additives [78].

The cellular antioxidant defense mechanism serves two primary functions: inhibiting the production of free radicals and deactivating them if they are generated. To achieve this, several enzymatic and non-enzymatic defense molecules have evolved to mitigate oxidative damage by transferring excess electrons during the detoxification process. Key antioxidant enzymes, such as superoxide dismutase, catalase, and glutathione peroxidase, are crucial in neutralizing reactive oxygen species (ROS) and protecting cellular components from oxidative stress. Superoxide dismutase (SOD) catalyzes the conversion of superoxide radicals into hydrogen peroxide and oxygen, thereby preventing the harmful reactions that the superoxide might cause. Catalase (CAT) then converts hydrogen peroxide into water and oxygen, mitigating potential oxidative harm. Glutathione peroxidase (GPx) reduces hydrogen peroxide and organic hydroperoxides to water and corresponding alcohols, using glutathione (GSH) as a substrate, thus protecting cell membranes and lipids from peroxidation [79].

Non-enzymatic antioxidants are vital components of the cellular defense system against oxidative stress. These molecules, which include vitamins C and E, GSH, flavonoids, and carotenoids, directly scavenge ROS and RNS to prevent cellular damage. Vitamin C, a water-soluble antioxidant, neutralizes free radicals in the aqueous compartments of cells and tissues, while vitamin E, a lipid-soluble antioxidant, protects cell membranes from lipid peroxidation. GSH, a tripeptide, is critical in maintaining the redox balance within cells and regenerating other antioxidants. Flavonoids and carotenoids in various fruits and vegetables also contribute to antioxidant defense by inhibiting oxidative reactions and stabilizing free radicals [17]. These non-enzymatic antioxidants complement enzymatic antioxidants, ensuring a comprehensive protection against oxidative damage and maintaining cellular health.

Increased oxidative stress and specific chemical stress are common features in ASD individuals [80,81]. The former hypothesis, suggesting that ROS/RNS plays a significant role in ASD, is now widely accepted as factual [82]. The role of ROS/RNS in ASD is not yet fully understood; however, the redox imbalance and oxidative stress are essential components of ASD pathogenesis. Several studies have found elevated levels of oxidative stress markers in individuals with ASD, indicating that they experience more severe oxidative damage compared to neurotypical individuals [83]. Autism is associated with increased lipid peroxidation markers such as blood lipid peroxides and thiobarbituric acid reactive compounds compared to neurotypical controls [84,85]. Some indirect markers, such as phospholipase A2 and the loss of membrane lipoprotein asymmetry, have previously been documented in ASD individuals, as mentioned in the review of Bjørklund et al. [83]. Furthermore, the ASD patients had elevated levels of pro-oxidants, such as perchlorethylene, hexane, and pentane, as well as heavy metals, such as mercury, lead, and arsenic. Autistic patients’ blood circulation showed elevated amounts of cytokines and xanthine oxidase, which can produce free radicals [83].

Individuals with ASD present low plasma and cellular GHS (L-γ-glutamyl-L-cysteinyl-glycine) levels and a lower GSH reserve capacity, increasing oxidative stress. A GSH redox imbalance is common in ASD children, as evidenced by lower quantities of reduced GSH, higher oxidized glutathione (GSSG), and a lowered GSH/GSSG redox ratio [86]. Usui et al. [81] found that a considerable reduction in cysteine glutathione disulfide in children with ASD is associated with impaired cysteine metabolism and increased oxidative stress. Major antioxidants such as GSH and cysteine are essential for fighting oxidative stress and sustaining cell health. GSH is vital in protecting cells against toxins, particularly in the brain. Antioxidant deficits, such as with GSH, aggravate symptoms in people with ASD, including behavioral abnormalities, cognitive impairments, and GI problems [83]. Moreover, ASD exhibits reduced activities of key antioxidant enzymes, specifically SOD and CAT [17,87]. Children with ASD have lower amounts of antioxidant serum proteins such as transferrin, which lowers free ferrous ion concentrations, and ceruloplasmin, which prevents the peroxidation of membrane lipids caused by metal ions like iron and copper [84]. 

Mitochondrial dysfunction is one of the leading causes of ASD pathology, since there are abnormalities in the mitochondrial electron transport system that occur in the brains of patients with ASD [23,88]. In addition, children with ASD present the inactivation of mitochondrial aconitase, an enzyme that helps catalyze citrate to isocitrate [86]. Mitochondrial dysfunction can contribute to altered energy metabolism. Mitochondria are essential for producing adenosine triphosphate (ATP), the energy currency of the cell, and any impairment in their function can lead to reduced energy availability for cellular processes. It has been reported that reduced ATP synthesis in the brains of individuals with ASD by magnetic resonance spectroscopy and elevated lactate and pyruvate levels have indicated a damaged energy and mitochondrial metabolism [89]. In addition, mitochondrial dysfunction can lead to an increased production of ROS, further exacerbating oxidative stress.

Oxidative stress is recognized as a factor associated with the development of ASD, and impairments in the antioxidant system can impact the function of the brain [90]. The brain is particularly vulnerable to oxidative stress, especially during early development [91]. This vulnerability can lead to neurodevelopmental disorders, including ASD. The brain’s high metabolic activity and oxygen consumption make it susceptible to damage from ROS. Individuals with ASD exhibit signs of oxidative stress in the brain [92]. This includes findings from studies that have reported physiological abnormalities such as increased lipid peroxidation and reduced antioxidant defenses in brain tissue derived from patients with ASD. Children with ASD often have reduced levels of key antioxidants and antioxidant enzymes in the brain, such as GSH, SOD, and CAT [93]. This reduced antioxidant capacity can impair the brain’s ability to counteract oxidative stress, leading to increased oxidative damage and contributing to the pathophysiology of ASD. The oxidative stress observed in the brains of individuals with ASD is associated with dysfunction in the brain regions involved in critical functions such as speech, auditory processing, social behavior, memory, and motor coordination [92]. This suggests that oxidative stress may play a role in the cognitive and behavioral symptoms associated with ASD. 

**Figure 2:**
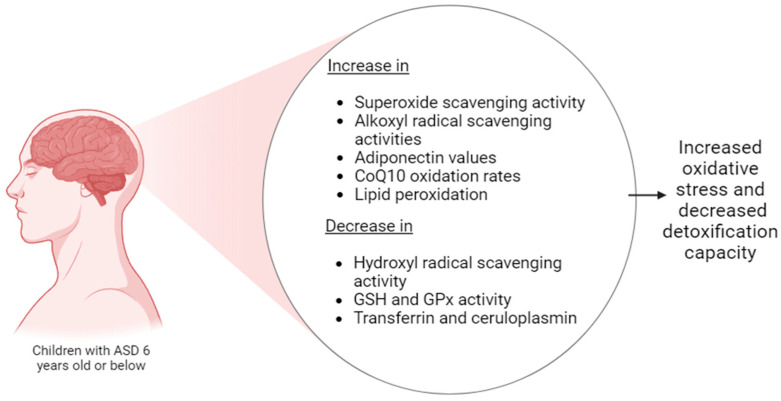
Diagram of affected activities in increased oxidative stress in children with autism spectrum disorder. Figure created by the authors.

Some studies state that oxidative stress in children with ASD may suggest an increase in this activity and a more complicated pattern involving different types of ROS and antioxidants [94]. Children with ASD have shown a decrease in hydroxyl radical scavenging activity and an increase in superoxide and alkoxyl radical scavenging activities. A study by Hirayama et al. [95] suggests that redox changes in children with ASD represent a more complex antioxidative shift. They also revealed that the CoQ10 oxidation and serum 8-hydroxy-2′-deoxyguanosine (8-OHdG) values in their ASD group, consisting of children >6 years old, were significantly increased compared to their control group, which translates into higher levels of oxidative stress and substantially higher adiponectin values in children with ASD of the same ages, as mentioned before. This study also suggests that oxidative stress in ASD patients is not simply increased but shows a complicated imbalance, including multiple types of ROS, subsequent ROS chain reactions, and comprehensive modifications in the antioxidant system. These affected factors are summarized in Figure 2.

Moreover, ASD impairs all players in the vitamin E/vitamin C/glutathione network, which controls oxidative stress. Both ASD rodent models and autistic people exhibit changes in the expression of necessary antioxidant enzymes of the ROS scavenging system in the brain and the peripheral blood [96]. It has been documented that the concentration of vitamin E in the blood was lowered and was connected with autism-like behaviors in autistic patients [97]. Vitamin C deficiency has been noted in children with ASD, with an increased prevalence of scurvy reported in this population [98]. This deficiency may be attributed to the malabsorption of vitamin C and/or a diet low in fruits and vegetables. Additionally, studies have shown that children with ASD have lower levels of vitamin C in their blood compared to healthy controls [99].

Oxidative stress can also stimulate our immune response and cause allergic reactions, such as food allergies, which, as mentioned above, are sometimes present in ASD children. Thus, this suggests that the innate antioxidant protection mechanism of patients with allergic reactions is less effective than that of a healthy individual [100].

Targeting oxidative stress in ASD comprises strategies to reduce ROS generation and improve antioxidant defenses. Dietary therapies with antioxidant-rich foods and supplementing with vitamins and minerals, including vitamins E, C, and selenium, have shown promise in reducing oxidative damage. Furthermore, lifestyle changes, such as minimizing exposure to environmental pollutants and regulating inflammation, are essential components of a complete strategy for managing oxidative stress in people with ASD. Understanding and managing oxidative stress is critical for improving the health and well-being of people with ASD. Current research is investigating viable therapies and therapy techniques to combat oxidative stress and its effects on autism.

## 4. Dietary Intervention in ASD

Dietary interventions such as probiotics, antioxidants, gluten-free and casein-free, and ketogenic diets have shown the potential to reduce GI discomfort, oxidative stress, and related behavioral issues in individuals with ASD. GI issues, which are prevalent in ASD, often exacerbate food selectivity, sensory sensitivity, and food neophobia. By addressing GI discomfort, these dietary interventions can positively impact sensory sensitivity, unhealthy food preferences, and food neophobia. Improved gut health often reduces sensory-related food aversions by minimizing the physical discomfort associated with eating, making it easier for individuals with ASD to accept a wider variety of foods. Furthermore, these interventions can help regulate mood and behavior, which are closely linked to mealtime experiences and food preferences. These dietary approaches can be integrated with gradual food exposure therapy and applied behavior analysis to further align with behavioral strategies. For instance, as GI symptoms are alleviated, caregivers and therapists can successfully introduce new foods using positive reinforcement techniques, encouraging individuals to try different food textures and flavors in a supportive environment. Combining dietary interventions with parental support and structured mealtime routines can help create consistent and positive food experiences, ultimately addressing the core challenges of food neophobia and sensory sensitivity in ASD. 

Moreover, many children with ASD face challenges related to being underweight or overweight/obesity due to food selectivity and dietary imbalances [10]. Some individuals prefer a limited range of foods, resulting in insufficient nutrients of dietary fiber, vitamins, calcium, iron, and potassium, while others favor calorie-dense foods rich in protein, carbohydrates, and fats, contributing to obesity [10]. Sensory sensitivities and feeding challenges further influence these weight extremes, underscoring the need for personalized nutritional care. Also, the evidence suggests that individuals with ASD are more prone to food allergies and intolerances, which often necessitate tailored dietary interventions [101]. 

Given the combined factors of oxidative stress, GI issues, allergies, and weight management challenges in ASD, it is essential to implement dietary interventions that regulate food intake while meeting the minimum nutritional requirements for this population. These approaches address physical health and support behavioral and sensory improvements, making them a critical component of ASD management.

### 4.1. Diet High in Antioxidants

As previously mentioned, inflammation and oxidative stress may significantly contribute to the pathogenesis and severity of ASD. Consequently, diets rich in antioxidants have been explored as potential interventions for managing symptoms in individuals with ASD, primarily due to their ability to mitigate oxidative stress by protecting against ROS’ harmful effects and preventing free radicals’ formation [102]. Certain plant dietary antioxidants, such as carotenoids, phenolics, flavonoids, and vitamins, have been shown to possess anti-inflammatory, antioxidant, and immune properties that could benefit neurological and GI health, thereby supporting better food tolerance and potentially mitigating sensory sensitivities and neophobia [86,103,104,105].

**Figure 3:**
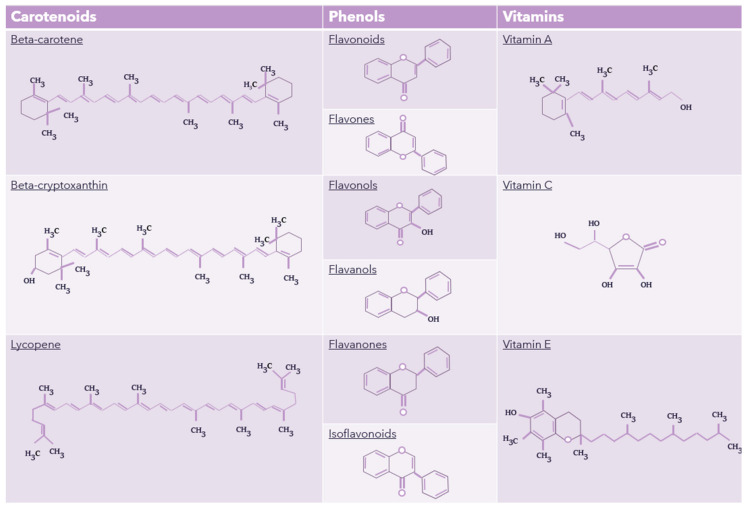
Chemical structures of carotenoids, phenols, and main antioxidant vitamins. Figure created by the authors.

In general, antioxidants have several bioactive properties; for example, anthocyanins reduce lipid peroxidation and have antimicrobial activity, beta-carotene lowers cardiovascular risk, beta-cryptoxanthin prevents osteoarthritis progression, and flavonoids offer antioxidant and anti-inflammatory benefits [106,107]. Lycopene supports mitochondrial homeostasis and intestinal health, while vitamins A, C, and E play critical roles in immune regulation, antioxidation, and neuroprotection [96,108,109,110]. These properties suggest that antioxidants may positively influence the health and well-being of children with ASD. The chemical structures of carotenoids, phenols, and significant antioxidant vitamins are depicted in Figure 3.

Carotenoids are fat-soluble pigments that give carrots, tomatoes, and pumpkins red, orange, and yellow colors. Many carotenoids have antioxidant and anti-inflammatory properties that may help alleviate ASD symptoms. For example, a study has shown that when beta-carotene derivatives are provided orally after birth to newborns from autism-prone mice families, they can prevent and/or improve autistic symptoms (reciprocal social interaction, repetitive grooming/bedding behavior), as well as the biochemical markers associated with ASD [111]. Recent findings show that carotenoids, such as lycopene, beta-carotene, lutein, astaxanthin, and fucoxanthin, have anti-apoptotic effects in neurodegenerative diseases. Their antioxidant properties and roles as signaling molecules and in gene regulation help reduce brain cell death, which may also impact autism [112]. In the same context, a study found that lycopene reduces propionic acid (PPA)-induced memory impairment and oxidative damage through its antioxidant and anti-inflammatory effects [113]. It protects serum and brain tissues from PPA-induced oxidative damage in rats by up-regulating the Nrf2/HO-1 pathway and down-regulating IL-1α, IL-8, TNF-α, and NF-κB levels. Lycopene may also improve memory, learning, and apoptosis regulation, showing potential therapeutic benefits in PPA-induced autism spectrum disorder [113].

Among carotenoids, astaxanthin, a lipid-soluble pigment in the terpene family, shows the most potent antioxidant potential [114]. Astaxanthin is naturally produced by the microalgae Haematococcus Pluvialis, and animals like salmon, shrimp, and crabs acquire astaxanthin by consuming these algae, leading to a red-orange pigmentation. Studies have shown astaxanthin’s potent anti-inflammatory and neuroprotective effects. For example, in a prenatal valproic acid-induced mouse model of ASD, astaxanthin improved behavioral disorders and oxidative stress [115]. Similarly, another study investigated the role of the renin–angiotensin system (RAS) and Notch signaling in ASD using a valproic acid rat model, as well as the therapeutic potential of astaxanthin. The results suggested that behavioral, histological, and molecular analyses showed that astaxanthin improved neuronal integrity, reduced inflammation, and modulated RAS, particularly enhancing the protective RAS arm and inhibiting harmful pathways. These results suggest that astaxanthin could be a promising treatment for ASD [116].

Polyphenols, including flavonoids, phenolic acids, anthocyanins, and stilbenoids, are natural compounds abundant in plants, fruits, and vegetables, known for their potent antioxidant and anti-inflammatory properties [117]. Their antioxidant properties help neutralize free radicals, reducing oxidative stress, often elevated in individuals with ASD [96]. Additionally, polyphenols’ anti-inflammatory effects may help mitigate neuroinflammation, a contributing factor in ASD symptoms, making them of interest for ASD-related dietary interventions [118].

Some flavonoids like quercetin and luteolin, found in many fruits and vegetables, exhibit potent antioxidant and anti-inflammatory properties, which may be beneficial in addressing neurodegenerative diseases and autism-related issues [119,120,121]. Tsilioni et al. [122] investigated the effects of a polyphenolic dietary intervention (with luteolin, quercetin, and rutin) on children with ASD who exhibited elevated serum levels of pro-inflammatory markers (IL-6 and TNF-α). The results showed that after 26 weeks of treatment, the phenolic formulation improved autistic behavior, and their serum levels of TNF-α and IL-6 significantly decreased compared to the study’s beginning due to the phenolic compounds’ anti-inflammatory properties. On the other hand, anthocyanins are a natural pigment that belong to the flavonoid family and are responsible for the red, purple, and blue colors in many fruits, vegetables, and flowers, such as blackberries, raspberries, grapes, cherries, and eggplants. In vitro studies have reported that anthocyanins could cross the blood–brain barrier and provide neuroprotection by inhibiting NF-kB translocation and reducing neuroinflammatory markers like TNF-α [123,124].

In a similar context, resveratrol, a polyphenolic stilbenoid with significant anti-oxidative and anti-inflammatory properties, was tested in animal models with ASD. For example, a study investigated the potential of resveratrol to reduce neuroinflammation in a rat model of ASD [125]. ASD-like symptoms were induced by infusing propanoic acid into the rats’ brains. Resveratrol was administered in varying doses (5–15 mg/kg/day) for four weeks, and the rats were tested for behavioral changes, such as social interaction, anxiety, and memory, as well as biochemical markers like oxidative stress, mitochondrial function, TNF-α, and MMP-9 levels. The results showed that resveratrol dose-dependently improved the rats’ behavioral, neurological, and biochemical deficits, suggesting that it could be a promising therapeutic agent for managing ASD symptoms by reducing oxidative stress, mitochondrial dysfunction, and inflammation. Another study has reported that resveratrol may reduce inflammation and immune dysfunction in BTBR mice, a model for autism, by decreasing the expression of cytokines (IL-6, TNF-α, IFN-γ) and the JAK1/STAT3 signaling pathway at both the mRNA and protein levels [126]. The study of Santos-Terra et al. [127] suggested that resveratrol prevented neuron loss in the hippocampus and altered interneuron populations on a valproic acid-induced autism model in rats. This provides promising therapeutic insights, but the role of antioxidants in managing ASD requires further clinical research to confirm these positive findings and fully understand their long-term benefits and practical applicability.

As previously mentioned, vitamins also play an important role in antioxidant activity. Vitamin A plays a crucial role in regulating the development of the central nervous system through its active metabolite, retinoic acid [128]. Retinoic acid is instrumental in promoting intestinal immunity and preserving the integrity of mucosal epithelial cells [129]. Research has demonstrated that vitamin A can increase oxytocin levels through the CD38 pathway in individuals with autism. Oxytocin, in turn, can potentially enhance brain activity and significantly improve the social abilities of children with autism [130]. Studies have reported that therapy with vitamin A can help alleviate symptoms associated with ASD [131].

Vitamin C, or ascorbic acid, is a hydrosoluble antioxidant that neutralizes and removes oxidant agents and recycles other antioxidants while acting as a potent reducing agent [128]. Additionally, vitamin C is a cofactor in the biosynthesis of neurotransmitters such as serotonin [132] and has been found to have mood-improving properties [133]. Children with ASD are likely to have low vitamin C values due to their diet being poor in fruits and vegetables due to low contact or malabsorption [99]. Clinical trials have aimed to restore the antioxidant network of vitamin C and glutathione in individuals with ASD [134]. In a double-blind, placebo-controlled study, the safety and efficacy of glutathione alone or combined with vitamin C and N-acetyl L-cysteine were evaluated in children with autism over eight weeks. Both treatments showed improvements in developmental skills and behavior compared to the placebo, with a positive correlation between behavior changes and the GSH/GSSG ratio. The evidence suggests that targeting this antioxidant network may improve ASD symptoms [96]. Vitamin E, or α-tocopherol, is a liposoluble antioxidant known for its ability to scavenge free radicals. Reduced levels of vitamin E have been observed in the blood of individuals with ASD, and its supplementation is associated with attenuating ASD-like symptoms [97]. 

Several studies suggest supplementing vitamins and minerals can help reduce ASD symptoms [135,136]. For example, Meguid et al. [137] supplemented zinc in 30 children with ASD, ages 3 to 8. The results of this study reduced Childhood Autism Rating Scale (CARS) scores, improved cognitive–motor performance, and lowered copper levels. In a study where vitamin A was supplemented in children with ASD, the symptoms of this disorder were significantly enhanced, suggesting that supplementation can also be a viable option for this population [130]. Similarly, Adams et al. [135] compared children aged 5–16 (*n* = 55) to nonsibling, neurotypical controls (*n* = 44) of a similar age, gender, and regional distribution. The results showed that oral vitamin and mineral supplementation helps children with autism improve their nutritional and metabolic state, including methylation, glutathione, oxidative stress, sulfation, ATP, NADH, and NADPH. This topic has been extensively discussed, as previously reviewed by Önal et al. [138], with an excellent table displaying the relevance of several studies in which vitamin and mineral supplementation was given to children with ASD.

In addition to plant-derived antioxidants, animal-derived antioxidants, such as specific proteins and peptides, also could play a significant role in dietary interventions for individuals with ASD. However, fewer studies have been conducted in this context than with plant antioxidants. Animal-derived antioxidants like carnosine, taurine, and glutathione are known for their ability to reduce oxidative stress and protect against cellular damage. Glutathione, a cysteine-glutamate-glycine tripeptide abundant in animal tissues, is one of the body’s most important antioxidants. It supports immune function, detoxification, and the regulation of oxidative stress, which has been linked to ASD-related behavioral and neurological symptoms [139]. An open-label pilot study by Radwan et al. [140] reported improved oxidative markers (e.g., lipid peroxides) of oral supplementation with Opitac™ glutathione in patients with ASD. However, this did not consistently correlate with clinical improvements in ASD symptoms. Glutathione supplementation was generally well tolerated, with stomach upset reported in four out of six subjects. The study highlights the need for further research. 

Taurine, an amino acid in animal products like meat and seafood, is an effective antioxidant. It is critical in modulating neurotransmission, calcium homeostasis, and inflammatory responses, key factors in ASD symptom management [141]. The review of Rubio-Casillas et al. [142] discusses the role of microglia in ASD and how their activation can lead to neuroinflammation and neurodevelopmental dysfunction. It highlights the potential of taurine, an amino acid with neuroprotective properties, to counteract neuroinflammation by shifting microglia from the harmful M1 to the beneficial M2 phenotype. Taurine may also aid in promoting neurogenesis and synaptogenesis and restoring autophagy by inhibiting the Akt/mTOR pathway, which is linked to excessive glutamate release and impaired synaptic pruning in ASD. The research suggests that taurine could reduce oxidative stress, inflammation, and behavioral symptoms in individuals with ASD. Additionally, taurine has been found to improve cognitive function and protect against neurotoxicity. The authors propose taurine as a potential treatment for ASD, suggesting further clinical trials to validate its therapeutic efficacy. 

Similarly, carnosine, a dipeptide found in meat and fish, has demonstrated neuroprotective and antioxidant effects. It could benefit individuals with ASD by reducing oxidative stress in the brain and improving cognitive function [143]. Clinical trials have shown some improvements in communication skills, behavior, and sleep in children with ASD following carnosine supplementation. For example, carnosine supplementation (800 mg/day for eight weeks) showed an improvement in receptive speech and social attention, a decrease in apraxia, and an overall increase in brain function [144], while the same dose for 10 weeks achieved a reduction in hyperactivity and non-compliance [145]. The supplementation of 500 mg/day for two months of carnosine reduced the incidence of sleep disturbance in children with ASD [146]. However, the results have been inconsistent, with some trials showing benefits and others not confirming these effects [147]. A meta-analysis of clinical trials concluded that the current evidence is insufficient to recommend carnosine supplementation as a treatment for ASD, indicating a need for further research [148].

### 4.2. Gluten-Free/Casein-Free Diet

Individuals with ASD frequently suffer from casein and gluten allergies, which have a substantial influence on dietary management, resulting in the restriction of certain foods [101,149]. Casein, a protein found in milk and dairy products, and gluten, a protein found in wheat, barley, and rye, are frequent allergens that can cause severe responses in susceptible people. A gluten-free diet eliminates foods containing wheat, barley, rye, and any products made from these cereals, such as flour, bread, pasta, and bakery items [150]. On the other hand, a casein-free diet entails avoiding dairy products such as milk, yogurt, cheese, butter, cream, and ice cream, among others [151].

In children with ASD, the immune system’s response to these proteins can aggravate both GI and behavioral symptoms [152]. Many studies indicate that people with ASD have a higher sensitivity or intolerance to casein and gluten [101,149]. According to the theories, the immunological and GI systems have a role in ASD development by modulating the gut–blood–brain barrier via bacterial byproducts such as lipopolysaccharides and short-chain fatty acids, which impact cytokine production. Furthermore, bacterial byproducts such as serotonin may influence neuropeptide production, whereas gluten and casein peptides are thought to promote opioid system activation. These neuropeptides may affect social behavior and communication, contributing to the ASD etiology. This sensitivity can cause various GI disorders, including bloating, constipation, diarrhea, and abdominal pain. Furthermore, these dietary proteins have been linked to initiating inflammatory responses, which are thought to contribute to the overall symptomatology of autism [152]. Children with ASD have greater levels of pro-inflammatory cytokines following exposure to dietary proteins from gluten and casein compared to controls [153].

By eliminating gluten and casein from the diet, individuals may experience fewer digestive issues, contributing to a more positive mealtime experience and reducing resistance to trying new foods. Additionally, minimizing GI discomfort may help reduce the sensory overload associated with eating, leading to an improved tolerance of various food textures, smells, and flavors. Case reports highlight the benefits of therapeutic diets for children with ASD, demonstrating improvements in eye contact and communication skills and the alleviation of symptoms like constipation and vomiting, mainly through the adoption of a GFCF (gluten-free, casein-free) diet [154]. The factors mentioned above and other theories suggest that children with ASD must pay particular attention to their diet. Various studies have shown that parents often choose a GFCF diet for their children with ASD, as there appears to be a relation between the mechanisms of action of the immune system and the GI system [50,155,156]. Parents also report increased behavioral issues, such as hyperactivity, difficulty focusing and sleeping problems, and GI problems when not following a GFCF diet [157]. 

There are multiple double-blinded studies where a GFCF diet was not significantly beneficial. Most of these studies monitor very few sample sizes, some examples ranging from 12 to 74 children with ASD of different ages and follow-up periods from 1 to 24 weeks [158,159,160,161,162,163]. In one study, participants were evaluated three times: before the intervention, after six months, and after 12 months of adhering to a GFCF. However, post-intervention assessments did not reveal significant changes in behaviors related to language, sociability, sensory speech communication, cognitive consciousness, autistic seclusion, or physical health disability [162].

Another study examined the impact of a gluten-free diet on a cohort of sixty-six children (aged 36–69 months) with ASD. The participants were divided into two groups: Group I followed a gluten-free diet, while Group II consumed at least one regular meal containing gluten daily for six months. Each child underwent a comprehensive behavioral and psychometric assessment at baseline and after the intervention. After six months, both groups showed improvements on the tests, but no significant differences were observed between the two groups [164]. However, some detailed studies with up to four years of follow-up for a gluten-free, casein-free (GFCF) diet have reported significant benefits for children with GI issues, primarily diarrhea and constipation. These studies found that the diet improved GI conditions and enhanced social behaviors and physiological symptoms compared to children with ASD who do not have GI issues [165,166]. 

Nevertheless, systematic reviews on GFCF suggest that the evidence is currently insufficient to either support or refute its effectiveness in alleviating ASD symptoms [167]. We can conclude that more studies with an increased sample size and prolonged follow-up periods are needed to conclude whether children with ASD benefit from this specific diet. However, this diet is the most commonly used in children with ASD [2].

### 4.3. Ketogenic Diet and Essential Fatty Acids

The ketogenic diet (KD), although less commonly used, is a promising treatment for neurological disorders; however, prospective (long-term observation) controlled trials with high sample sizes are required to establish official recommendations. KD consumes high amounts of fatty foods, low carbohydrates, and adequate protein [168]. By consuming low amounts of carbohydrates, our organism is forced to use ketone bodies as a fuel source, providing energy to the brain [169]. The ketogenic diet is beneficial and improves ASD symptoms related to socio-affective deterioration, as well as fear or nervousness, decreasing their characteristic behaviors [170]. This dietary approach can create a more conducive environment for reducing food selectivity and neophobia over time by stabilizing neurological and gastrointestinal function.

This neuroprotective diet is often recommended for neurological disorders that are neurodegenerative or that have a metabolic defect, for instance, Alzheimer’s disease, Parkinson’s disease, migraines, etc., to lessen symptoms, and it has shown overall cognitive improvements [171]. For this reason, it also has been considered a complementary treatment to ASD due to its correlation with epilepsy [168]. Epilepsy is a common neurological comorbidity associated with ASD that affects the brain by exhibiting seizures. It is caused by different factors or categories such as genetics, structural, metabolic, infectious, immune, and others whose etiology remains vague [172]. Epilepsy affects about 1 out of 10 people with ASD [173].

In a pilot follow-up study, 30 children with ASD followed a ketogenic diet for six months. Seven subjects could not tolerate the diet, and five others adhered for only one or two months. The remaining subjects reported significant improvements in overall autistic behaviors, achieving a greater capacity for concentration and learning [174]. In a similar study, 45 children with ASD, aged 3 to 8 years, participated in a controlled treatment divided into three groups: one group followed a ketogenic diet, another group followed a GFCF diet, and the control group followed a balanced diet. The results showed improved CARS (Childhood Autism Rating Scale) scores for the ketogenic and GFCF diets compared to the control group. The ketogenic diet group also improved cognition and sociability, specifically the ability to perceive and interpret other people’s social behavior, compared to the GFCF and control groups, suggesting the potential benefits of these diets; however, more studies are needed to draw definitive conclusions [175]. Additionally, the ketogenic diet may act as an antioxidant, as ketone bodies are known to inhibit the production of ROS at a mitochondrial level by increasing NADH oxidation [176]. 

The pilot study of Mu et al. [169] investigated the relationship between behavioral parameters, blood metabolites, and trace elements in 10 typically developed controls and 17 children with ASD, both at baseline and after three months of treatment with a modified KD regimen. The key findings demonstrated that, at baseline, children with ASD had higher quantities of galactose intermediates, gut microbe-derived trimethylamine N-oxide, and N-acetylserotonin, but lower levels of 3-hydroxybutyrate and selenium than the control group. After three months on the KD regimen, the ASD group’s circulating ketone and acetylcarnitine levels increased, while selenium levels returned to control values. The study also discovered a new unfavorable association between selenium levels and behavioral ratings. High responders to the KD intervention had higher concentrations of 3-hydroxybutyrate and ornithine and lower galactose levels, which improved our understanding of the metabolic alterations in ASD and the potential benefits of KD intervention.

Supplementation for fatty acids, specifically omega 3 and 6, is also a popular treatment in children with ASD since these contribute to brain function, neurotransmission, and the composition of cell membranes and help decrease inflammation and oxidative stress. Omega 3 and omega 6 are dietary polyunsaturated fatty acids. Omega 3 is derived from alpha-linolenic acid that turns into a 20–22 chain of carbon atoms that can convert to eicosapentaenoic acid (EPA) and docosahexaenoic acid (DHA) once metabolized. Omega 6 derives from linoleic acid and turns into a longer chain; however, it converts to arachidonic acid instead [177].

Fatty acids have been used as a treatment in multiple neurological disorders, such as schizophrenia [178], depression [179], and bipolar disorder, amongst other disorders like ASD [180]. In children with ASD, DHA and EPA levels have been recognized to be significantly lower than in the general population [181]. Therefore, supplementation for these fatty acids has shown benefits in decreasing ASD-like symptoms and helping with overall social behavior and repetitive behaviors [148,182]. Moreover, according to a study by Matthews and Adams [2], this diet usually improves GI problems, attention issues, language/communication, and depression, among others.

### 4.4. Probiotic-Rich Diet

Probiotic dietary interventions have gained increasing attention as a potential therapeutic approach for individuals with ASD, given the growing evidence linking gut health to neurological function through the gut–brain axis [23]. As mentioned above, many individuals with ASD experience GI issues such as constipation, diarrhea, and abdominal pain, which can exacerbate behavioral symptoms and negatively impact quality of life. Probiotics, which consist of live beneficial bacteria, can help restore balance in the gut microbiota, reduce inflammation, enhance gut integrity, and promote the production of beneficial metabolites, such as short-chain fatty acids, which play a role in brain health and behavior, offering a promising avenue for dietary intervention in ASD [18]. These improvements in gut health can alleviate discomfort, potentially making the sensory experience of eating less overwhelming, thereby reducing food selectivity and encouraging the acceptance of a broader range of foods. Studies suggest probiotics may provide a potential alternative to restrictive nutritional interventions because they can help reconstruct or stabilize the intestinal barrier via increased mucin production and fortified tight junctions, synthesize antioxidants to protect the gut from pathogens, produce digestive enzymes, and modulate immune responses and suppress dysregulated immune functions [183]. 

Some clinical studies have explored the effects of probiotics on individuals with ASD. A survey by Shaaban et al. [65] found that a mixture of probiotic strains (*Bifidobacterium longum*, *Lactobacillus rhamnosus*, *Lactobacillus acidophilus*; 1 × 10^8^ CFU/g; 5 g/day) supplementation over three months led to improvements in the fecal microbiota of ASD children (explicitly increasing the levels of Bifidobacteria and Lactobacilli compared to baseline), in GI symptoms, and in certain behavioral aspects, such as reduced irritability and hyperactivity. In another study, 30 children with ASD were intervened upon with exclusion diets (gluten- and casein-free) and a six-week prebiotic diet using Bimuno^®^ galactooligosaccharide (B-GOS^®^) to evaluate GI symptoms, gut microbiota, and behavior [184]. The B-GOS^®^ prebiotic intervention, combined with exclusion diets, significantly improved behavioral traits in the children, mainly by reducing anti-sociability scores. While no significant changes in GI symptoms were observed with the B-GOS^®^ intervention alone, its combination with exclusion diets led to reduced abdominal pain, improved bowel movements, and changes in gut microbiota composition, including lower levels of *Bifidobacterium* spp. and *Veillonellaceae*, and higher levels of *Faecalibacterium prausnitzii* and *Bacteroides* spp. Similarly, probiotic supplementation containing *Lactobacillus* (60%), two strains of *Bifidumbacteria* (25%), and one strain of *Streptococcus* (15%) was given orally three times a day for four months to 10 autistic children (2–9 years), their nine non-autistic siblings (5–17 years), and 10 non-autistic children (2–11 years), as a control. The results showed that probiotics normalized the Bacteroidetes/Firmicutes ratio, *Desulfovibrio* spp., and the amount of Bifidobacterium spp. in the feces of autistic children and reduced their intestinal inflammation [55].

When considering dietary interventions for individuals with ASD, it is equally important to identify which diets or food components should be avoided. One of the most recommended dietary restrictions, as was stated before, is the avoidance of gluten and casein, as many individuals with ASD are thought to have sensitivities or intolerances to these proteins. Highly processed foods, often rich in additives, preservatives, and artificial colors, should also be minimized, as these can potentially trigger hyperactivity or increase sensory sensitivities in some individuals [185]. Additionally, diets high in sugars and unhealthy fats may contribute to weight imbalances and metabolic issues in those with ASD [34]. Therefore, they should be limited to promoting overall health and well-being. Avoiding these dietary elements can help reduce gastrointestinal discomfort, improve behavior, and support better nutritional outcomes in individuals with ASD.

## 5. Conclusions and Future Directions

ASD is a condition that requires increased attention due to its growing prevalence. Addressing ASD necessitates a multidisciplinary approach, considering the numerous factors involved. Children with ASD often experience metabolic and GI symptoms that can be derived from the condition itself. While many details remain to be discovered and understood, parents have turned to dietary interventions to alleviate behavioral and GI symptoms. Diets such as ketogenic, high-antioxidant, and casein/gluten-free diets have garnered interest in managing ASD symptoms. The initial research suggests they may offer potential benefits. The exclusion of casein and gluten is based on the hypothesis that these components might trigger inflammatory or neurochemical responses in some individuals with ASD. These diets typically focus on reducing carbohydrate consumption and increasing the intake of healthy fats and lean proteins, potentially positively impacting brain function and energy metabolism. However, the evidence supporting the effectiveness of these dietary exclusions in children with ASD remains limited, necessitating further research to validate their widespread use. Ketogenic diets may increase the risk of deficiencies in essential vitamins such as A, D, and E. Therefore, children on such diets must receive appropriate medical supervision and, when necessary, supplements to ensure healthy growth and development. Regarding the supplementation of vitamins, minerals, and antioxidants, while beneficial, excessive intake can lead to pro-oxidant effects, potentially causing harm. Therefore, any supplementation should be discussed with the child’s physician to consider all aspects of their care.

In conclusion, while specific diets may offer benefits for some children with ASD, parents need to work closely with medical and nutritional professionals to ensure these diets are safe, balanced, and appropriate for the individual needs of each child. Dietary approaches in ASD children have shown improvements in attention span, cognition, and irritability, suggesting that following a specific diet improves behavioral aspects. However, given individual differences, not all diets will elicit the same response in every child, emphasizing the need for personalized assessment by a nutritionist or specialist. Further research is required to understand these treatments’ long-term effectiveness and applicability in managing ASD in children.

The limitations of a narrative review include the potential for selection bias, where studies with positive outcomes may be favored, while those with inconclusive or negative results might be excluded. This issue is often better addressed in meta-analyses, which use more systematic methods to avoid such a bias. The observational nature of many of the included studies complicates the ability to establish causality, and the variability in methodologies, such as small sample sizes or a lack of proper controls, may affect the validity of the findings. Additionally, results from specific populations may not be easily generalized to those with different cultural or socioeconomic backgrounds. Finally, due to the heterogeneity of ASD, it is challenging to generalize the effectiveness of specific dietary interventions across the entire ASD population. These limitations should be considered to improve the quality and relevance of future reviews.

## Figures and Tables

**Table 1:** Studies related to food selectivity in children with ASD.

Food/Characteristic Studied	Population Number (*n*)	Methodology	Important Results	Reference
Fruits, Vegetables, Dairy/milk, Flours, Fats, Legumes, Meats, Sweets, Snacks	105	An “Aut–Eat questionnaire” survey was administered to parents of 105 children with ASD and 95 neurotypical children regarding 137 items or foods regularly consumed by their children.	Neurotypical children ate a significantly greater variety of food groups than children with ASD. Toddlers with ASD ate more snacks than neurotypical toddlers; older children with ASD ate significantly fewer snacks than neurotypical children of the same age.	[47]
Fruits, Fruit juice, Vegetables, Starchy vegetables, Unrefined carbohydrates, Refined carbohydrates, Eggs, Raw meats, Processed meats, Meat alternative, Dairy	325	Online surveys of parents and caregivers of children (ages 3 to 16) who were neurodivergent	The level of food acceptance varies in response to different food attributes, such as color, presentation, temperature, and texture. The food attributes preferred are crunchy, smooth, moist, and soft foods, with refined carbohydrates being the most accepted group.	[44]
Grains, Fruit, Vegetables, Dairy, Fats, Sugars	39	24 h reminder with parents of 39 children aged 2 to 17 with ASD	Compared to the diet of neurotypical children, the diet of children with ASD has fewer grains and vegetables and greater amounts of sugar in their diet.	[46]
Rice, Refined carbohydrates, Fruits, Vegetables	68	68 surveys of parents of children with ASD aged 2 to 11 years and older	Children with ASD prefer easy to chew foods, such as rice and bread. They also prefer junk food and some fruits and vegetables. Because they have sensory problems, they do not like crunchy food and show an aversion to trying new food.	[45]
Grains and starches, Vegetables and marine food, Fruits, Meats, Foods rich in proteins, Dairy, Fat and sweets, Snacks	130	Survey of 130 parents who may have more than one neurodivergent child.	Children with ASD like foods that are high in sugar and fat. Children eat snacks more than once a day. Parents of children are interested in developing more nutritious snacks that are high in protein and low in calories with a minimum of additives.	[51]
Attributes of food such as texture, taste, color, and temperature	173	Questionnaire to parents about their child with ASD to determine the consumption of a variety of foods according to different attributes (color, texture, etc.) and preferences	The average group of children with ASD preferred foods with a certain appearance, disliked sticky foods, preferred crispy foods and sweet foods, and refused foods with mixed ingredients.	[9]
Animal products, Plant products, Bakery products, Fats, Milk, Fruits, Vegetables, and Others	62	46 children with ASD and 16 non-ASD children were included in this study to find a correlation between ASD children’s diet and gut microbiota. Parents participated in mealtime questionnaires.	Children with ASD showed lower values of docosahexaenoic acid, docosapentanoic acid, iron, copper, iodine, and vitamins K, B6, and C.	[48]
